# Arthrose post-traumatique carpo-métacarpienne du cinquième doigt traitée par arthroplastie stabilisée: à propos de deux cas et revue de la littérature

**DOI:** 10.11604/pamj.2018.30.163.12445

**Published:** 2018-06-22

**Authors:** Wassim Zribi, Mohamed Zribi, Mohamed Ben Jemaa, Wajdi Bouaziz, Ameur Abid, Abdesslem Naceur, Yosr Hantati, Moez Trigui, Zoubayer Ellouze, Kamel Ayedi, Hassib Keskes

**Affiliations:** 1 Service de Chirurgie Orthopédique et Traumatologique, CHU Habib Bourguiba de Sfax-Tunisie; 2 Service de Radiologie, CHU Hédi Chaker de Sfax-Tunisie

**Keywords:** Fracture-luxation carpo-métacarpienne, arthrose post-traumatique, arthroplastie, arthrodèse, Carpo-metacarpal fracture and dislocation, post-traumatic arthrosis, arthroplasty, arthrodesis

## Abstract

Les fractures-luxations anciennes de la base du cinquième métacarpien peuvent se compliquer d'une arthrose post-traumatique gênante sur le plan fonctionnel et de prise en charge difficile. La technique d'arthroplastie stabilisée comporte une résection arthroplastique de la base du cinquième métacarpien associée à une arthrodèse diaphyso-métaphysaire latérale entre le quatrième et le cinquième métacarpien. A travers deux observations, nous présentons les objectifs de cette technique d'arthroplastie stabilisée et ses particularités par rapport aux autres techniques destinées au traitement des séquelles des fractures-luxations de la base du M5.

## Abstract (en)

Old fractures and dislocations of the base of the fifth metacarpal may result in post-traumatic arthrosis which is troublesome from a functional point of view and characterized by a difficult management. Stabilized arthroplasty is based on arthroplastic resection of the base of the fifth metacarpal associated with lateral diaphysometaphyseal arthrodesis between the fourth and the fifth metacarpal. This study involving two patients aimed to describe the advantages and the peculiarities of stabilized arthroplasty compared to other techniques in the treatment of the sequelae of fractures and dislocations of the base of the fifth metacarpal.

## Introduction

Les fractures-luxations du cinquième rayon de la main représentent une entité fréquente en traumatologie de la main [1]. La réduction en urgence avec stabilisation par embrochage reste le meilleur traitement avec le plus souvent de bons résultats fonctionnels [2]. Des arthroses post-traumatiques peuvent être observées avec un retentissement fonctionnel important. Ces séquelles sont l'apanage des fractures-luxations négligées, d'une mauvaise réduction initiale ou d'une communition articulaire importante rendant difficile toute restitution anatomique de la surface articulaire [3-5]. Les objectifs du traitement chirurgical des fractures-luxations anciennes du cinquième rayon sont la restauration de la longueur du cinquième métacarpien (M5), la suppression du conflit articulaire carpo-métacarpien et la conservation de la mobilité du 5^ème^ rayon [6]. La technique d'arthroplastie stabilisée répond théoriquement à ces trois objectifs.

## Patient et observation

**Figure 1:**
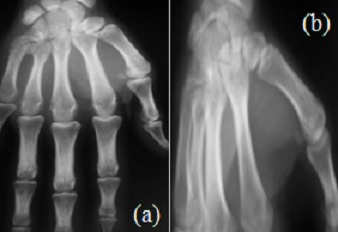
Radiographie de la main droite (A: incidence de face, B: incidence oblique) fracture-luxation dorso-latérale négligée de la base du M5

Nous rapportons deux cas d'arthrose post-traumatique de la base du M5. Les deux patients consultaient pour des douleurs résiduelles en regard de la base du M5 avec une gêne fonctionnelle importante et diminution de la force de serrage limitant les gestes de la vie quotidienne. Le premier patient, âgé de 22 ans, droitier, a été vu au quatrième mois d'une luxation négligée de la base du M5 droit faisant suite à un coup de poing contre un plan dur (Figure 1). Le deuxième patient, âgé de 39 ans, droitier, était victime d'une fracture-luxation de la base du M5 ayant consolidé en cal vicieux articulaire avec un raccourcissement de M5 du fait d'une mauvaise réduction initiale. Une arthroplastie stabilisée du M5 a été pratiquée chez les deux patients.

**Figure 2:**
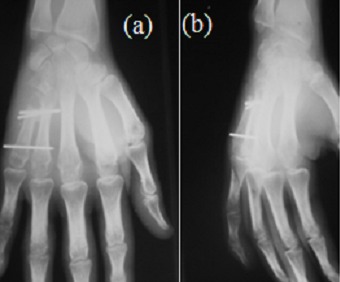
Radiographie de la main droite (A: incidence de face, B: incidence oblique) résection arthroplastique de la base du M5 et arthrodèse métaphyso-diaphysaire des deux derniers métacarpiens stabilisée par embrochage transversal

**Description de la technique opératoire:** La chirurgie se déroule sous anesthésie locorégionale avec hémostase préventive par garrot pneumatique à la racine du membre. L'incision est longitudinale située à mi-distance entre le 4^ème^ et le 5^ème^ métacarpien. L'exposition de la base du M5 permet de visualiser les dégâts ostéo-cartilagineux. Une ostéotomie métaphysaire proximale du M5 permet de réséquer 5 à 10 mm de la région épiphyso-métaphysaire et d'éliminer le conflit articulaire carpo-métacarpien du 5^ème^ rayon. Les surfaces corticales médiale du 4^ème^ métacarpien (M4) et latérale du M5 sont avivées à la scie sur 2 cm au niveau de la région métaphyso-diaphysaire proximale. Le fragment osseux réséqué initialement de la base du M5 est préparé après avoir enlevé tous les débris cartilagineux. Il est ensuite chassé et intercalé au niveau de la région avivé entre M4 et M5. La stabilisation se fait par un embrochage transversal entre M4 et M5 de façon parallèle sous contrôle d'un amplificateur de brillance et en maintenant le 4^ème^ et le 5^ème^ doigt en syndactylie pour ne pas avoir des troubles rotatoires ou une abduction du 5^ème^ rayon (Figure 2).

**Figure 3:**
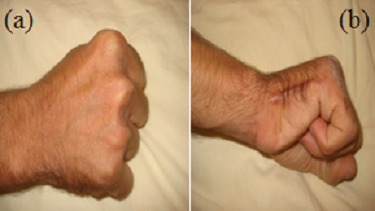
Aspect clinique de la main au dernier recul de face (A) et de profil (B) cicatrice opératoire de bonne qualité avec une fonction du serrage quasi normale du 5ème rayon

**Figure 4:**
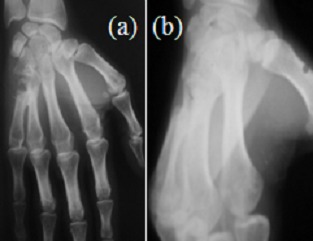
Aspect radiologique de la main au dernier recul (A: incidence de face, B: incidence oblique) bonne consolidation M4-M5

La fermeture se fait après hémostase et lavage abondant à fin d'éliminer tout débris ostéo-cartilagineux résiduel. Une manchette plâtrée est confectionnée en fin d'intervention maintenue pendant 6 semaines associée à une syndactylie entre le 4^ème^ et le 5^ème^ doigt. Une mobilisation digitale est débutée immédiatement après la chirurgie. L'ablation du plâtre se fait entre 6 à 8 semaines suivant le contrôle radiologique de l'arthrodèse. L'ablation des broches se fait à 2 mois suivie d'une rééducation régulière des chaînes digitales et du poignet avec renforcement de la force d'opposition et du serrage. Après vérification radiologique de la consolidation et une rééducation bien suivie, les deux patients ont repris leur travail à 3 mois post-opératoires. A quatre ans de recul, le résultat fonctionnel a été jugé très bon avec une mobilité normale du 5^ème^ doigt notamment en abduction, adduction, une conservation de la longueur et de la force de serrage par rapport au côté controlatéral. La mobilité en flexion-extension du bloc M4-M5 est de 25° comparée à 40° de l'autre côté (Figure 3). L'opposition pouce-cinquième doigt est symétrique. Le bilan radiologique montre une arthrodèse bien consolidée dans les deux cas (Figure 4).

## Discussion

Les fractures-luxations du M5 peuvent se compliquer de séquelles arthrosiques avec gêne fonctionnelle importante suite à un retard de prise en charge ou par un défaut de réduction initiale [2, 3]. Elles peuvent être associées à des douleurs au niveau de l'interligne carpo-métacarpien du 5^ème^ rayon, une perte de la force du serrage, d'une perte de la mobilité carpo-métacarpienne en flexion et d'un raccourcissement du cinquième rayon avec défaut d'abduction. Un bilan radiologique avec des clichés bien réussis de face et de profil strict de la main complété si nécessaire par une tomodensitométrie, permet une meilleure analyse de ces séquelles. Plusieurs techniques ont été proposées dans la littérature pour améliorer la fonction de la main [1, 3, 5]. La réduction secondaire donne de bons résultats même au-delà de 3 mois en dehors d'une communition articulaire importante [3, 7, 8]. L'arthrodèse carpo-métacarpienne du 5^ème^ rayon a été le traitement de choix pour la plupart des auteurs [2, 5, 9, 10]. Elle permet de rétablir la longueur de M5 avec disparition du conflit et une fonction correcte de la main. La force de serrage est le plus souvent récupérée environ dans 80% des cas. Une certaine mobilité en flexion extension est conservée par transfert de la mobilité dans l'articulation hamato-pyramidale aux pris d'une sollicitation accrue de l'interligne hamato-pyramidale et risque d'évolution vers l'arthrose intra-carpienne [11]. A.C *Masquelet* et col indiquent cette arthrodèse d'emblée lors de la prise en charge initiale pour une communition importante épîphyso-métaphysaire de la base du M5 avec des lésions chondrales majeures [12]. La résection arthroplastique avec interposition tendineuse ou prothétique ne permet pas de rétablir la longueur en cas de raccourcissement important [13].

L'arthroplastie stabilisée a été proposée par *Th. Dubert* en 1994 avec description bien précise de ses étapes chirurgicales [6]. En répondant aux objectifs de la prise en charge des séquelles des fractures-luxations anciennes de la base du M5, cette technique pallie aux défaillances techniques des autres procédures chirurgicales. D'une part, elle supprimer le conflit interne par résection de la base du M5. Une résection de 5 à 10 mm est possible sans fragiliser l'insertion de l'extenseur ulnaire du carpe. D'autre part, elle rétablit et stabilise la longueur du M5 par arthrodèse diaphyso-métaphysaire latérale M4-M5. Après avivement cortical, l'espace inter-métacarpien est comblé par des greffons spongieux. L'ostéosynthèse est assurée par des vis ou des broches transversales. L'utilisation de mini-plaque permet de bien stabiliser l'arthrodèse et de diminuer le délai d'immobilisation. Entre autre, cette technique conserve la mobilité du cinquième rayon par transfert à l'interligne carpo-métacarpien du 4^ème^ rayon obligatoirement indemne et dont la physiologie est très proche de celle du 5^ème^ rayon bien que son secteur de flexion extension soit plus faible de 50% [6, 14]. Le diagnostic des fractures-luxations de la base du M5 doit être précoce avec une exploration systématique devant tout traumatisme centrée sur le 5^ème^ rayon en insistant sur des incidences radiologiques bien réussites de face et de profil strict de la main. La tomodensitométrie permet de mieux analyser les dégâts ostéo-cartilagineux en cas de communition importante. Le meilleur traitement des fractures-luxations de la base du M5 reste chirurgical avec réduction initiale de la surface articulaire. La chirurgie à ciel ouvert reste le meilleur garant pour répondre au cahier de charge d'une chirurgie articulaire initiale. Une mauvaise prise en charge initiale expose au risque de séquelles d'arthrose post-traumatique carpo-métacarpienne. Nous pensons que cette technique est originale et trouve sa supériorité dans le traitement de ces séquelles. Cependant, elle nécessite une rigueur dans sa réalisation afin d'éviter l'évolution vers la pseudarthrose de l'arthrodèse qui peut être plus gênante et douloureuse qu'une articulation congruente et arthrosique.

## Conclusion

Les fractures-luxations anciennes de la base de la cinquième métacarpienne compliquées d'arthrose peuvent être gênantes sur le plan fonctionnel. Le traitement dépend de l'importance des dégâts ostéo-cartilagineux et de l'ancienneté des lésions. L'arthroplastie stabilisée trouve sa supériorité par rapport aux autres techniques permettant le rétablissement la longueur du cinquième métacarpien, la suppression du conflit articulaire carpo-métacarpien avec un transfert de la mobilité à l'articulation carpo-métacarpienne du quatrième rayon.

## Conflits d’intérêts

Les auteurs ne déclarent aucun conflit d'intérêts.
